# Differential Distribution of *Salmonella* Serovars and *Campylobacter* spp. Isolates in Free-Living Crows and Broiler Chickens in Aomori, Japan

**DOI:** 10.1264/jsme2.ME17183

**Published:** 2018-03-29

**Authors:** Masashi Okamura, Miyuki Kaneko, Shinjiro Ojima, Hiroki Sano, Junji Shindo, Hiroaki Shirafuji, Satomi Yamamoto, Taishi Tanabe, Yasuhiro Yoshikawa, Dong-Liang Hu

**Affiliations:** 1 Laboratory of Zoonoses, Kitasato University School of Veterinary Medicine Towada, Aomori Japan; 2 Laboratory of Wildlife Science, Kitasato University School of Veterinary Medicine Towada, Aomori Japan; 3 Subtropical Disease Control Unit, Division of Transboundary Animal Diseases, Kyusyu Research Station, National Institute of Animal Health, National Agriculture and Food Research Organization Chuzan, Kagoshima Japan; 4 Laboratory of Microbiology, Kitasato University School of Veterinary Medicine Towada, Aomori Japan; 5 Faculty of Risk and Crisis Management, Chiba Institute of Science Choshi, Chiba Japan

**Keywords:** *Salmonella*, *Campylobacter*, crows, broilers

## Abstract

*Salmonella* and *Campylobacter* cause foodborne enteritis mainly via the consumption of raw/undercooked contaminated poultry meat and products. Broiler flocks are primarily colonized with these bacteria; however, the underlying etiology remains unclear. The present study was conducted in order to obtain further information on the prevalence and genotypic distribution of *Salmonella* and *Campylobacter* in free-living crows and broiler flocks in a region for 2 years, thereby facilitating estimations of the potential risk of transmission of *C. jejuni* from crows to broiler flocks. *Salmonella* serovars Bredeney and Derby were isolated from 8 and 3 out of 123 captured crows, respectively, both of which are not common in broiler chickens. *Campylobacter* were isolated from all 89 crows tested and *C. jejuni* was prevalent (85 crows). Pulsed field gel electrophoresis showed broad diversity in the crow isolates of *C. jejuni*. However, 3 crow isolates and 2 broiler isolates showing similar banding patterns were assigned to different sequence types in multi-locus sequence typing. These results indicate that crows do not share *Salmonella* serovars with broilers, and harbor various genotypes of *C. jejuni* that differ from those of broiler flocks. Thus, our results indicate that crows are not a potential vector of these bacteria to broiler flocks in this region.

*Salmonella* and *Campylobacter* are the most common bacterial causes of human foodborne enteritis worldwide (21). These infections are mainly associated with the consumption of raw/undercooked contaminated poultry meat and products (33, 37, 41). In order to control human infection, countermeasures need to be simultaneously implemented at all stages of poultry production from farm to table (20, 34). Due to the high prevalence of *Salmonella* and *Campylobacter* in broiler flocks, the primary target to decrease the incidence of these infections is pre-harvest control at the farm level, which includes enhanced biosecurity to avoid the transmission of bacteria from the environment to bird flocks on the farm.

Potential reservoirs of *Salmonella* and *Campylobacter* have been identified or presumed, such as personnel, water supply, feed, transport crates, and proximal farms growing other livestock or poultry (1, 19, 24, 26, 43, 45). Wild animals have also been suggested as vectors of infections in poultry (14, 45). Recent studies provided the potential attribution of wild birds to *Salmonella* and *Campylobacter* infections in humans (8, 29). Wild birds living in close proximity to broiler farms were found to carry *Salmonella* spp. and *Campylobacter* spp. (10). Among wild birds, crows are generally known to breed in urban and agricultural settings and fly around their roosts and locations such as livestock farms at which they forage. Thus, if crows carry these bacteria, they may serve as a potential reservoir or carrier of *Salmonella* and *Campylobacter* to broilers.

The present study investigated the prevalence of *Salmonella* and *Campylobacter* in crows and their genetic relatedness with isolates from broilers by comparisons of pulsed field gel electrophoresis (PFGE) patterns and multi-locus sequence typing (MLST) in order to demonstrate the potential transmission of bacteria between crows and broiler flocks.

## Materials and Methods

### Sample collection

This study was conducted in Aomori prefecture, the most northeastern part of the main island of Japan. This region thrives with agriculture and animal husbandry. A total of 123 crows were captured on 19 occasions between October 2012 and April 2013 and 13 occasions between October 2013 and April 2014. Crows were captured using box traps placed at two sites 2.2 km from a city in Aomori prefecture. This area is semi-rural, and more than 20 broiler farms as well as a few scattered cattle and pig farms exist within a radius of 20 km from the trap sites. After being captured, crows were transferred to the Wildlife Quarantine Facility in the Kitasato University School of Veterinary Medicine. The species of crows were visually confirmed by the size and shape of the beaks. They consisted of 78 jungle crows (*Corvus macrorhynchos*), 28 carrion crows (*C. corone*), and 17 unidentified crows. The weights of the captive crows ranged between 340 and 770 g. All 123 crows were tested for *Salmonella*, of which 89 were also tested for *Campylobacter* (Table 1). The collection of wild animals for scientific investigation in this study was approved by the Prefectural governor of Aomori prefecture (Prefecture Directive No. 13) based on the Protection and Control of Wild Birds and Mammals and Hunting Management Law (Act No. 88 of 2002). Crows were euthanized by the inhalation of isoflurane followed by adjunctive exsanguination according to the AVMA Guidelines for the Euthanasia of Animals (2). The ceca were taken aseptically and divided into 2 parts of approximately 0.4 g each; one for the isolation of *Salmonella* and another for *Campylobacter*. Sex and age (adult or nestling) were presumed at necropsy by the presence of testes/ovaries and the bursa of Fabricius, respectively. The sex of seven crows was not established because of the immaturity of their genital organs.

### Isolation of *Salmonella* spp

Cecal samples were homogenized with a 9-fold volume of Hajna tetrathionate broth (Eiken, Tokyo, Japan) and incubated at 41.5°C for 24 h for enrichment. A loopful of each homogenate was then streaked on desoxycholate-hydrogen sulfate-lactose agar plates. After an incubation at 37°C for 24 h, black-colored colonies assumed to be *Salmonella* were then serotyped based on the Kauffmann-White scheme using a commercially available agglutination test (Denka Seiken, Tokyo, Japan) with O and H antigens.

### Isolation of *Campylobacter* spp

Cecal samples were homogenized with *Campylobacter* enrichment medium (25) and incubated at 37°C for 48 h in a microaerobic environment for enrichment. A loopful of each homogenate was streaked on modified charcoal cefoperazone deoxycholate agar plates (Oxoid, Basingstoke, UK). After an incubation at 37°C for 48 h in a microaerobic environment, white-colored colonies assumed to be *Campylobacter* isolates were identified at the species level using multiplex PCR (53).

### PFGE analysis

Isolates of *Salmonella* and *Campylobacter* were subtyped by PFGE following the PulseNet protocol (https://www.cdc.gov/pulsenet/pathogens/pfge.html). Genomic DNA was digested with *Xba*I (Takara, Shiga, Japan) for *Salmonella* and *Sma*I (Takara) for *Campylobacter*. PFGE banding patterns were analyzed using Fingerprint II software (Bio-Rad Laboratories, Hercules, CA, USA) with the Dice coefficient with 1.0% band tolerance and 1.0% optimization and the unweighted pair group method with arithmetic averages (UPGMA). The *Salmonella* serovar Braenderup H9812 genome digested with *Xba*I was used as the standard for both bacteria. Isolates collected from broilers in another study conducted in 2011–13 (Okamura *et al.*, unpublished data) were also included in the analysis.

### MLST analysis

MLST was performed as described (12). Genomic DNA was purified using a DNeasy Blood & Tissue kit (Qiagen, Düsseldorf, Germany). The amplification reactions for 7 housekeeping genes were performed with 7.5 pmol of each primer using KOD-Plus (Toyobo, Osaka, Japan). PCR products were gel-purified, mixed with the respective sequencing primers, and sequenced on a 3730XL DNA Analyzer using BigDye Terminator v3.1 Cycle sequence kit (Applied Biosystems, Foster city, CA, USA) following the manufacturer’s instructions. Sequence data were collated and alleles assigned using the *Campylobacter* MLST database (http://pubmlst.org/campylobacter/). ST9010 was newly submitted for ST designation following the procedure described on the *Campylobacter* MLST website. The assignment of STs to CCs was taken from the *Campylobacter* MLST database. The MLST profiles of these isolates are shown in the supplemental table.

### Antimicrobial susceptibility test

The minimal inhibitory concentrations of ampicillin (ABPC), amoxicillin (AMPC), kanamycin (KM), nalidixic acid (NA), norfloxacin (NFLX), and enrofloxacin (ERFX) for *C. jejuni* isolates were obtained using an agar dilution method, in accordance with the Clinical Laboratory Standards Institute guidelines (7).

## Results and Discussion

### Prevalence of *Salmonella*

*Salmonella* spp. were isolated from the ceca of 11 out of 123 crows (8.9%). Eight and 3 isolates were identified as serovars Bredeney and Derby, respectively (Table 2). Serovar Bredeney rarely causes human infection with contaminated food such as poultry (32) and other products such as peanut butter (50). Serovar Derby is one of the prevalent serovars found in pigs in North America (36, 52). In Europe, *S.* Derby is one of the most prevalent serovars in slaughter pigs, and also ranks among the ten most frequently isolated serovars in humans (16). In Japan, this serovar has rarely been isolated from pigs (11, 47) and human cases (13), and to the best of our knowledge, these serovars have not been isolated from broiler flocks. One of the most frequently isolated serovars from broilers has generally been serovar Infantis (3). Therefore, *Salmonella* does not appear to be transmitted between crows and broiler flocks, but may provide a potential link between crows and pig farms in this area.

### Prevalence of *Campylobacter*

*Campylobacter* spp. were isolated from all 89 crows tested (100%). *C. jejuni*, *C. lari* and other unidentified *Campylobacter* were isolated from 85 (95.5%), 9 (10.1%), and 9 crows (10.1%), respectively, and the 2 species were simultaneously detected from 14 crows (Table 3). A previous study reported that wild birds harbored *Campylobacter* spp. in Denmark, wild birds on livestock farms carried *C. jejuni* at a high frequency, and the highest carriage rate of 61.8% was found in thrushes (23). In the Mid-Atlantic region of the US, the overall prevalence of *C. jejuni* was 7.2%, with the highest being crows (23%) and gulls (25%) (27). Weis *et al.* more recently reported that *Campylobacter* was isolated from 66.9% (85/127) of free-living American crows (*C. brachyrhyncos*) in California (51). The present study showed that the prevalence of *Campylobacter* was 100%, and *C. jejuni* (85/89) was predominant in crows. Since captured juvenile crows carried *Campylobacter*, even nestlings may be exposed to bacteria in roosts or their surroundings (46). *C. jejuni* is generally isolated more frequently than *C. coli* from broiler chickens (15). In the present study, *C. coli* was not isolated, while *C. lari* was detected albeit at a low prevalence. In Canada, *C. lari* was isolated from river water (66%) and waterfowl (32%), and one isolate from a seagull showed 100% homology to another from river water (49). Since there is a river within the 2-km radius of the box traps in the present study, free-living crows may also drink river water that may be contaminated with *C. lari*.

### Genetic subtypes of *Salmonella* and *Campylobacter* isolates

All 8 isolates of *S.* Bredeney were from crows captured at 5 different sampling occasions between October 2012 and March 2013; nevertheless, they showed an identical PFGE pattern (data not shown). This result indicates the clonal distribution of this serovar in the environment of this region, supported by Cormican *et al.* (9) who demonstrated high similarity in the PFGE patterns of 97 out of 112 isolates of serovar Bredeney from various sources such as humans, poultry, bovine, and deer. Three isolates of *S.* Derby were not subtyped due to a smeared band pattern (data not shown).

**Fig 1:**
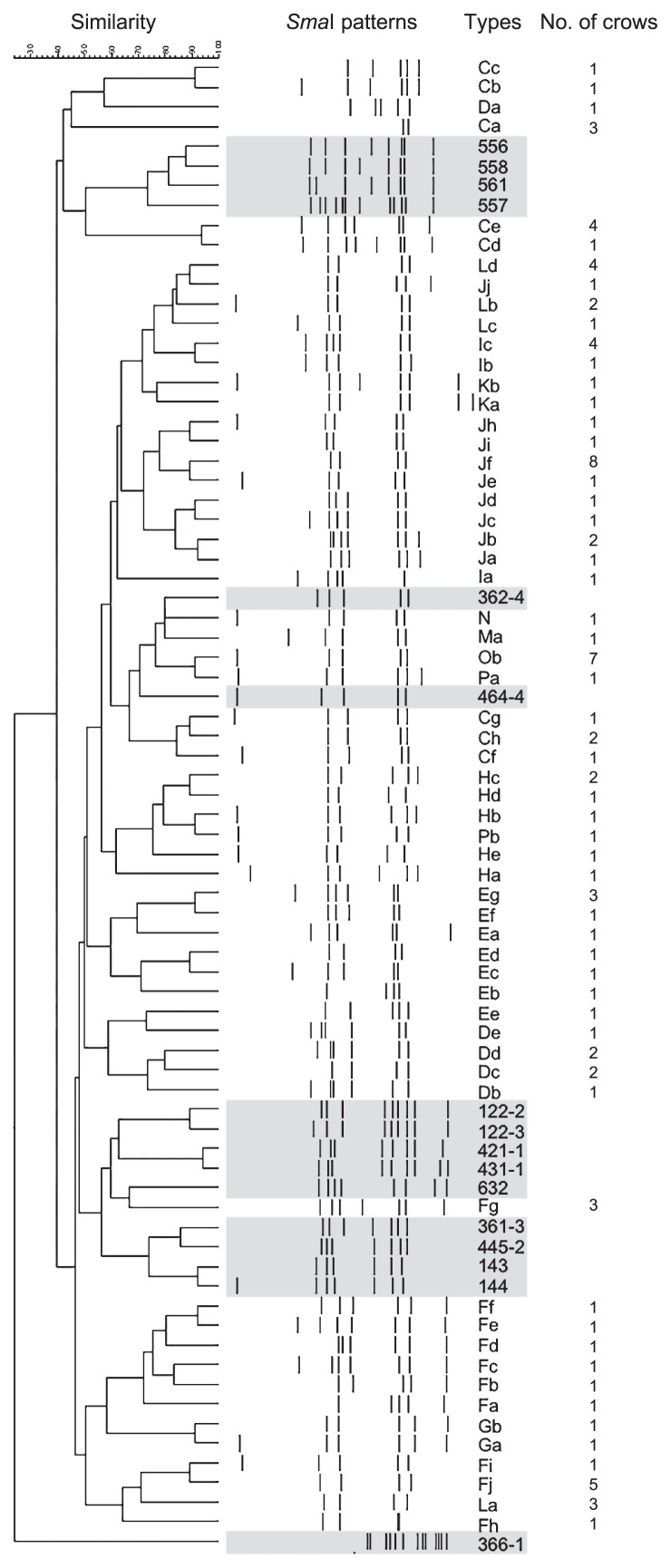
Dendrogram of *Campylobacter jejuni* isolates collected from crows in this study generated by pulsed-field gel electrophoresis (PFGE) patterns with *Sma*I. The isolates collected from broilers in the other study in 2011–13 were also included and shown in shaded area. The numbers of crows from which the respective PFGE patterns were isolated were also represented.

*C. jejuni* isolates showed a broad diversity of PFGE patterns, which was consistent with previous findings (40). Fig. 1 includes all 59 patterns of crow isolates in the present study and 16 patterns of isolates from broiler flocks in our surveillance between 2011 and 2013 (Okamura *et al.*, unpublished data). Each of the 43 different PFGE patterns originated from a single crow, while the other 16 patterns were isolated from 2–8 crows. This result suggests that crows obtain *Campylobacter* in diverse locations, and the roosts in which these crows congregate may be potential reservoirs retaining multiple strains of *Campylobacter*. Ten PFGE patterns (Ca, Dc, Dd, Fj, Ic, Jb, Jf, La, Ld, and Ob) were isolated at more than 2 different sampling occasions with an interval of 4 months for the pattern Ic and 11–15 months for the others. *Campylobacter* is known to survive against an atmospheric level of oxygen and/or poor nutrient supply (5), which may explain the persistence of the bacteria not only in poultry houses and farms (17, 44), but also in natural environments including crow roosts. This result implies that reservoirs are maintained for up to 1 year in crow roosts or their surroundings and may continuously expose juvenile and adult crows to different strains of *C. jejuni*.

In the PFGE analysis, 80% similarity was found between patterns Ma, N, and Ob from 9 crows and 362-4 from broilers and between Cf from crows and 464-4 from broilers. These isolates appeared to be “closely related” epidemiologically according to the Tenover criteria (48). However, some difficulties are associated with accurately interpreting differences in or the similarity of banding patterns using a PFGE analysis due to the lack of reproducibility and its application to an unsuitable context (4, 48). In MLST for the further analysis of genetic relatedness, isolates with the similar PFGE patterns of Ma, N, and Ob from crows and 362-4 and 464-4 from broilers were assigned to different types: ST2761, ST9010, ST1540, ST22, and ST3727, respectively (Table 4). Crow isolates with the PFGE pattern of Cf were not recovered from frozen stock and not analyzed by MLST. ST2761 was included in the ST-952 complex. This complex was previously reported to be predominant among crow isolates (39) and has been detected in rabbits, environmental water and soil, and free-living birds (28). In the *Campylobacter* PubMLST database, ST1540 is associated with wild birds and environmental water. It belongs to the ST-1275 complex, which was also identified in environmental water samples in Canada (30) and New Zealand (6). These isolates possibly originated from or are related to environmental water, which is supported by the isolation of *C. lari* in the present study as described above. ST9010 was newly identified in the present study, submitted, and added to the PubMLST database. ST22 isolates from broilers belong to the ST-22 complex. The isolates in this clonal complex have predominantly been detected from human stools, and sometimes from chickens, cattle, cow milk, and other sources, but not from wild birds (PubMLST). The isolates with ST3727 (the ST-45 complex) have been found in chickens in Japan, except for two from human stools in New Zealand and Sweden (PubMLST), while the ST-45 complex is one of the most common clonal complexes with broad host ranges, a so-called “generalist” (22, 42).

The antibiotic resistance profiles of these 5 isolates were elucidated (Table 4). Crow isolates with ST1540 and ST9010 showed markedly greater susceptibility to ABPC and AMPC than the other isolates, suggesting that these 2 isolates are crow-associated *C. jejuni* that have not been exposed to ABPC or AMPC. However, ST2761, which was also isolated from crows, is resistant to ABPC and AMPC, compared to the broiler isolates. This may be due to its potential origin from different livestock such as pigs and cattle treated with these antibiotics. A possible explanation is that these antibiotics are extruded via multidrug efflux mechanisms. In the CmeABC system, for example, not only a broad range of antibiotics and bile salts, but also heavy metals, dyes, and detergents may serve as its substances (31, 35, 38), which are ubiquitous in the environment. Thus, the results of the PFGE and MLST analyses and antibiotic resistance profiles suggest that free-living crows harbored various types of *C. jejuni* and did not share common lineages with broiler flocks.

*C. lari* isolates represented 7 PFGE patterns, each of which was isolated from 1–2 crows (data not shown). The isolation of *C. lari* and *C. jejuni* from river water, although not attempted in the present study, may allow us to compare their PFGE patterns, providing epidemiological information on *Campylobacter* spp. in the environment and wildlife.

Free-living crows harbored *Salmonella* serovars Bredeney and Derby with low prevalence and *C. jejuni* with high prevalence in the region of the present study. A wide diversity of genotypes of *C. jejuni* appears to persist in various environmental reservoirs. Free-living crows and broilers do not share *Salmonella* serovars or the same lineages of *C. jejuni*. We conclude that free-living crows do not serve as a vector transmitting *Salmonella* serovars and *C. jejuni* between the natural environment and broiler farms in crow habitats. *Salmonella* serovars and *Campylobacter* spp. distribute in free-living crows and broilers in different manners.

Further investigations on the prevalence of these bacteria in pigs and cattle, which are also known to harbor *S.* Derby, as described above, and *C. jejuni*, respectively, and the characterization of isolates will provide a better understanding of the potential role(s) of crows in the distribution of *Salmonella* serovars and *C. jejuni* between poultry, livestock, and wild birds.

## Supplementary Material



## Figures and Tables

**Table 1:** Summary of crows captured in the present study.

	*Corvus macrorhynchos*			*Corvus corone*			Unidentified		
			
	Male	Female	N.D.*	Male	Female	N.D.	Male	Female	N.D.
Juvenile	26 (23)**	27 (26)	1 (1)	1 (1)	0	2 (1)	8 (8)	3 (3)	4 (4)
Adult	10 (7)	14 (8)	0	12 (2)	13 (3)	0	0	2 (2)	0

Total	36 (30)	41 (34)	1 (1)	13 (3)	13 (3)	2 (1)	8 (8)	5 (5)	4 (4)
			
78 (65)		28 (7)						17 (17)	

* Not determined.

** All 123 captive crows were tested for *Salmonella*, of which 89 were also tested for *Campylobacter* spp. in parentheses.

**Table 2:** Prevalence of *Salmonella* serovars in crows.

	*Corvus macrorhynchos*			*Corvus corone*			Unidentified		
			
	Male	Female	N.D.*	Male	Female	N.D.	Male	Female	N.D.
Juvenile	Derby 1**	Bredeney 1 Derby 1	—	—	—	—	Bredeney 1	Derby 1	Bredeney 2

Adult	Bredeney 1	—	—	Bredeney 1	Bredeney 1	—	—	—	—

* not determined;

** the number of *Salmonella*-positive crows.

**Table 3:** Prevalence of *Campylobacter* spp. in crows.

	*Corvus macrorhynchos*			*Corvus corone*			Unidentified		
			
	Male	Female	N.D.*	Male	Female	N.D.	Male	Female	N.D.
Juvenile	*jejuni* 21**	*jejuni* 25	—	*jejuni* 1	—	—	*jejuni* 8	*jejuni* 3	—
	*lari* 4	*lari* 1					*lari* 1	*lari* 1	
	Others 3	Others 2							

Adult	*jejuni* 6	*jejuni* 8	*jejuni* 1	*jejuni* 2	*jejuni* 3	*jejuni* 1	—	*jejuni* 2	*jejuni* 4
	*lari* 1	Others 2			*lari* 1			*lari* 1	
	Others 1								

* not determined;

** the number of *Campylobacter*-positive crows.

**Table 4:** MLST types and antimicrobial susceptibility profiles of crow and broiler isolates showing similar PFGE patterns.

ID of isolates	PFGE patterns	ST	Clonal complex	Minimal inhibitory concentration (μg mL^−1^)					

				ABPC	AMPC	KM	NA	NFLX	ERFX
Crow									
62217	Ma	2761	ST-952 complex	8	8	8	4	0.25	≦0.06
62221	N	9010	Unassigned	0.125	≦0.06	4	4	≦0.06	≦0.06
62220	Ob	1540	ST-1275 complex	0.125	0.25	4	4	0.25	≦0.06

Broiler									
62218	362	22	ST-22 complex	2	4	8	4	0.25	≦0.06
62219	464	3727	ST-45 complex	2	2	4	8	1	≦0.06
