# Supplementary material for: Differential Distribution of Salmonella Serovars and Campylobacter spp. Isolates in Free-Living Crows and Broiler Chickens in Aomori, Japan
Source: Microbes Environ. 2018 Mar 29;33(1):77–82. doi: 10.1264/jsme2.ME17183 (PMC5877346; doi:10.1264/jsme2.ME17183)
Supplement: Supplementary file 1 [file 33_77_s1.pdf]

Supplemental table. MLST profiles of the *C. jejuni* isolates from crows and broilers with similar PFGE patterns.

ID of Isolates	PFGE patterns	ST	Clonal complex	MLST profiles						
				aspA	glnA	gltA	glyA	pgm	tkt	uncA
Crow										
62217	Ma	2761	ST-952 complex	18	22	72	98	205	301	16
62221	N	9010	-	64	288	71	189	43	109	60
62220	Ob	1540	ST-1275 complex	76	33	22	104	43	109	31
Broiler										
62218	362	22	ST-22 complex	1	3	6	4	3	3	3
62219	464	3727	ST-45 complex	48	7	10	4	183	7	1
